# Characterization of a bifunctional alginate lyase as a new member of the polysaccharide lyase family 17 from a marine strain BP-2

**DOI:** 10.1007/s10529-019-02722-1

**Published:** 2019-08-16

**Authors:** Guiyuan Huang, Shunhua Wen, Siming Liao, Qiaozhen Wang, Shihan Pan, Rongcan Zhang, Fu Lei, Wei Liao, Jie Feng, Shushi Huang

**Affiliations:** 1 Guangxi Key Laboratory of Marine Natural Products and Combinatorial Biosynthesis Chemistry, Guangxi Academy of Sciences, Nanning, China; 2 Research and Development Department, Xiamen Innodx Biotech Co. Ltd, Xiamen, China; 3 National Engineering Research Center for Non-Food Biorefinery, State Key Laboratory of Non-Food Biomass and Enzyme Technology, Guangxi Key Laboratory of Bio-refinery, Guangxi Academy of Sciences, Nanning, China; 4 College of Life Science and Technology, Guangxi University, Nanning, 530004 Guangxi China; 5 The Food and Biotechnology, Guangxi Vocational and Technical College, Nanning, China; 6 School of Pharmaceutical Science, Guangxi Medical University, Nanning, 530021 China

**Keywords:** Marine strain, Bifunctional alginate lyase, Polysaccharide lyase family 17, Alginate oligosaccharides, Bioenergy

## Abstract

**Objectives:**

Bifunctional alginate lyase can efficiently saccharify alginate biomass and prepare functional oligosaccharides of alginate.

**Results:**

A new BP-2 strain that produces alginate lyase was screened and identified from rotted *Sargassum*. A new alginate lyase, Alg17B, belonging to the polysaccharide lyase family 17, was isolated and purified from BP-2 fermentation broth by freeze-drying, dialysis, and ion exchange chromatography. The enzymatic properties of the purified lyase were investigated. The molecular weight of Alg17B was approximately 77 kDa, its optimum reaction temperature was 40–45 °C, and its optimum reaction pH was 7.5–8.0. The enzyme was relatively stable at pH 7.0–8.0, with a temperature range of 25–35 °C, and the specific activity of the purified enzyme reached 4036 U/mg. A low Na^+^ concentration stimulated Alg17B enzyme activity, but Ca^2+^, Zn^2+^, and other metal ions inhibited it. Substrate specificity analysis, thin-layer chromatography, and mass spectrometry showed that Alg17B is an alginate lyase that catalyses the hydrolysis of sodium alginate, polymannuronic acid (polyM) and polyguluronic acid to produce monosaccharides and low molecular weight oligosaccharides. Alg17B is also bifunctional, exhibiting both endolytic and exolytic activities toward alginate, and has a wide substrate utilization range with a preference for polyM.

**Conclusions:**

Alg17B can be used to saccharify the main carbohydrate, alginate, in the ethanolic production of brown algae fuel as well as in preparing and researching oligosaccharides.

## Introduction

Using brown algae as a biomass feedstock to produce third-generation fuels has attracted public and scientific attention in recent years (Enquist-Newman et al. 2014; Ji et al. 2016; John et al. 2011). Brown algae are a large group of multicellular marine algae that contain many lignin-free carbohydrates, grow quickly, and are not a primary food crop. Therefore, using brown algae as feedstock for biofuels does not face the same food issues as land-based biomass production (John et al. 2011; Jang et al. 2012; Kim et al. 2011). Directly converting the main polysaccharide constituents in brown algae into bioethanol is more competitive than producing ethanol derived from terrestrial plants. This approach is therefore one potential means of solving the energy crisis and future environmental problems (Enquist-Newman et al. 2014; Ji et al. 2016; Wargacki et al. 2012).

Alginate, mannitol, laminarin, and trehalose are the main carbohydrates in brown algae, among which alginate is the most abundant, accounting for approximately 50% (w/w) of the total sugar. Alginate is the most abundant polysaccharide in marine organisms and is the world’s second most abundant biopolymer after cellulose; mannitol is ranked second in algal carbohydrates, with a content of 20% (w/w) in some brown algae (Ji et al. 2016).

At present, industrial microorganisms used to produce ethanol cannot directly utilize the main components of brown algae, such as alginate, mannitol, laminarin, and fucose, as substrates for producing bioethanol. However, some wild-type microorganisms, such as the marine bacterium *Zymobacter palmae* can directly convert mannitol to alcohol (Okamoto et al. 1993). *Pichia angophorae* can simultaneously utilize mannitol and laminarin polysaccharides to produce alcohol (Horn et al. 2000). Some strains can also utilize dextran, glucose and mannitol to produce ethanol, which are released from brown algae after pretreatment with acids or enzymes (Adams et al. 2009; Lee and Lee 2012). Zhang et al. (2014) reported a strain that can directly utilize alginate to produce ethanol. To date, only a few wild strains have been reported to simultaneously utilize the main components of brown algae, including alginate, mannitol, laminarin, fucose and glucose, as carbon sources to produce ethanol (Ji et al. 2016). In addition, via genetic engineering methods, a strain was constructed that could produce ethanol using alginate as a carbon source (Takeda et al. 2011) and another strain was constructed that simultaneously used mannitol and alginate in seaweed (Enquist-Newman et al. 2014; Wargacki et al. 2012). This development provided a new alcohol production method using the main sugar in brown algae.

Alginate is the major structural component of the brown algal cell wall and is a linear polysaccharide composed of (1,4)-linked β-d-mannuronic acid (M) and α-l-guluronic acid (G), arranged in a homopolymeric (MM- or GG-blocks) or heteropolymeric random sequence (MG-or GM-blocks) (Tang et al. 2009). Since alginate cannot be directly used as a prospective biomass for bioethanol production by ethanol-producing microorganisms, hydrolysing alginate into monosaccharides is a critical step in directly converting alginate into ethanol (Kim et al. 2012). Two treatments for alginate saccharification in industrial processes are treatment with acids or alkali and treatment with alginate lyases. Compared with acidic or alkaline treatment, enzymatic treatment is considered a mild reaction and involves no fermentation inhibitors or environmental pollution. Therefore, isolating specific microorganisms that secrete alginate lyases with high activity is essential for efficiently saccharifying brown seaweed.

Alginate is degraded by the enzymolysis of a group of enzymes that catalyse the β-elimination of the 4-O-linked glycosidic bond to form unsaturated uronic-acid-containing oligosaccharides (Gacesa and Goldberg 1992). Based on their catalytic characteristics, alginate lyases can be divided into endolytic and exolytic lyases (Lee et al. 2012). By decomposing the glycoside bonds in the polymer of alginate, the endolytic alginate lyase releases unsaturated di-, tri- and tetrasaccharides as the main products. Exolytic alginate lyase further degrades oligomeric alginate into unsaturated monuronic acid (Lee et al. 2012). Based on substrate specificity, endolytic alginate lyases are divided into mannuronate lyases (polyM lyase, EC 4.2.2.3) and guluronate lyases (polyG lyase, EC 4.2.2.11). Dual-function lyases that catalyse both polyG and polyM have also been reported (Rahman et al. 2012). Furthermore, alginate lyases fall into three categories based on molecular mass: the 20–35 kDa, 35–40 kDa, and 40–60 kDa classes (Osawa et al. 2005). Based on amino acid sequence similarities, alginate lyases are classified into PL5, 6, 7, 14, 15, 17 and 18 families within 28 families of polysaccharide lyases (http://www.cazy.org/Polysaccharide-Lyases.html). Most alginate lyases with endolytic activity can hydrolyse sodium alginate (SA) to produce oligosaccharides and are classified into the PL5, 6, 7, 14, 17 and 18 families (Wong et al. 2000). Partial alginate lyases were reported to have exolytic activity and degrade alginate into monosaccharides (mannuronate or guluronate). These lyases were classified into the PL7 (Yagi et al. 2016), PL13 (Li et al. 2015), PL15 (Ochiai et al. 2006) and PL17 family (Kim et al. 2012).

Alginate metabolic pathways have been reported in *Sphingomonas* sp. A1 (Takase et al. 2010), in which the alginates are depolymerized into oligosaccharides, including disaccharides, trisaccharides, and tetrasaccharides, by three endolytic alginate lyases, A1-I, A1-II, and A1-III, in the cytoplasm. The oligosaccharides are then saccharified into monomers by exolytic alginate lyases A1-IV, and the monomeric sugars are finally converted to 4-deoxy-l-erythro-5-hexoseulose uronic acid (DEH) and used by the cells. Alginate is also directly monomerized to DEH by exolytic alginate lyases. Kim et al. (2012) reported an exotype oligoalginate lyase of the PL17 family, Alg17C, which degrades the alginate oligomers into monomeric sugar acids (i.e., DEH). Alg17C is considered a key enzyme for forming alginate monomers for the use of alginate as biomass for biofuel or chemical production (Kim et al. 2012).

Alginate oligosaccharides (AOS) are non-immunogenic, non-toxic and biodegradable polymers with many important bioactivities, such as antioxidant, antipathogenic, anti-inflammatory and anti-endoplasmic reticulum stress effects (Guo et al. 2016). The tri-, tetra-, penta- and hexasaccharides formed from the enzymatic degradation of SA promoted lettuce seedling growth (Iwasaki and Matsubara 2000), and pentasaccharide exhibited significant antitumour effects in osteosarcoma patients following surgery (Chen et al. 2017). Furthermore, alginate lyases will be useful tools for generating active AOS, medical treatment, and energy bioconversion (Li et al. 2011; Han et al. 2015).

In a previous study on converting brown algae to ethanol fuel, we isolated and screened a new bacterial strain that produced alginate lyase, temporarily named BP-2. In this study, the alginate lyase produced by strain BP-2, Alg17B, was isolated, purified and characterized. Further study showed that Alg17B not only degraded SA but was also bifunctional in hydrolysing both polymannuronic acid (polyM) and polyguluronic acid (polyG). Furthermore, Alg17B functioned as an endolytic and exolytic alginate lyase to hydrolyse SA, polyM, and polyG to produce alginate monomers as well as oligosaccharides with DP of 2–6. The present study may provide a theoretical basis for further studying the production of bioactive AOS and ethanol fuel via enzymatic hydrolysis of alginate.

## Materials and methods

### Strains and culture conditions

A strain producing alginate lyase was screened and identified from rotted *Sargassum* collected from Weizhou Island, Beihai, Guangxi Province, China, and provisionally named BP-2. Only analytical-grade chemical reagents were used in this study. The optimized fermentation medium was K_2_HPO_4_ 1.05%, KH_2_PO_4_ 0.45%, MgSO_4_·7H_2_O 0.2%, FeSO_4_·7H_2_O 0.001%, (NH_4_)_2_SO_4_ 0.6%, NaCl 2%, and SA (Aladdin, Shanghai, China) 0.4% (w/v). The culture conditions were pH 8.0 at 35 °C, a shaker speed of 200 rpm, an inoculum volume of 1% (v/v), a loading volume of 200 mL in a 500 mL conical flask, and a culture time of 24 h.

## 16S rDNA identification of the strain

BP-2 strain genomic DNA was used as the template. The primers for 16S rDNA amplification were 27f (5′-AGAGTTTGATCCTGGCTCAG-3′) and 1541R (5′-AAGGAGGTGATCACCC-3′). The polymerase chain reaction (PCR) conditions were 95 °C for 5 min; 95 °C for 1 min, 57 °C for 1 min, and 72 °C for 1 min 20 s for 30 cycles; 72 °C for 5 min. The PCR products were submitted to SinoGenoMax Co., Ltd. (China) for sequencing.

### Purification and activity assay of Alg17B

The fermentation broth was centrifuged at 7000 rpm for 30 min at 4 °C, and the supernatant was collected and freeze-dried for concentration. The concentrate was dialysed and desalted against a 20 mM pH 7.5 phosphate buffer at 4 °C. The desalted crude enzyme solution was further freeze-dried and concentrated. The sample was finally dissolved in 20 mM phosphate buffer (pH 7.5) and centrifuged at 7000 rpm for 30 min at 4 °C. The supernatant was obtained as the crude enzyme solution.

The crude enzyme solution obtained from the concentration was loaded onto a DEAE Sepharose Fast Flow (Kayon, Shanghai, China) column, which was pre-equilibrated with 20 mM pH 7.5 phosphate buffer for ion exchange chromatography. The column volume was 40 mL (1.5 cm × 40 cm), and the sample loading volume was 1 mL. NaCl gradient elution was performed at a rate of 1 mL/min. The fractions with alginate lyase activity were collected using an automatic fraction collector. The active fractions were concentrated, desalted and stored at − 20 °C for later use. Chromatography, concentration, and desalination were all performed at 4 °C.

The protein concentration was determined using the Bradford method (Marion 1976). Enzyme activity was measured by the Preiss method (Preiss and Ashwell 1962). 1 mL of double-distilled water and 0.5 mL of enzyme solution (0.1 mg/mL) were added to 1 mL of 0.2% (w/v) SA solution (0.2 g SA dissolved in 100 mL pH 7.5, 50 mM Tris–HCl buffer) to start the reaction. After incubating in a water bath at 37 °C for 30 min, the reaction was terminated by boiling the water bath, and the absorbance at 235 nm was measured after cooling. Under this condition, an absorbance increase of 0.01 per min was defined as an enzyme activity unit. The purified alginate lyase was concentrated and desalted using an ultrafiltration centrifuge tube (Millipore, Merck, Germany) with a molecular weight cut-off of 10 kDa, followed by sodium dodecyl sulphate polyacrylamide gel electrophoresis (SDS-PAGE) according to the method of Laemmli (1970).

### Effects of temperature on Alg17B activity and stability

Using 0.2% (w/v) SA as a substrate at pH 7.0, the effects of temperature on the purified Alg17B were investigated at 25 °C, 30 °C, 35 °C, 40 °C, 45 °C, and 50 °C to determine the optimal reaction temperature of the alginate lyase. Additionally, the purified enzyme solutions were incubated at above different temperatures for 1 h, rapidly cooled to 0 °C, and residual activities were measured under the standard assay conditions to evaluate the thermal stability of the enzyme.

### Effects of pH on Alg17B activity and stability

Using 0.2% (w/v) SA as a substrate, the effects of pH on Alg17B activity were measured by incubating the purified enzyme solutions in 200 mM Na_2_HPO_4_-citrate buffer at pH 7.0–8.5 under standard test conditions. Furthermore, the purified enzyme was respectively conserved in above different pH buffers at 4 °C for 24 h in advance, and residual activities were tested to estimate the pH stability of Alg17B.

### Effects of metal ions, surfactants and NaCl concentration on Alg17B activity

Using 0.2% (w/v) SA as a substrate, the effects of different metal ions and different surfactants on Alg17B activity were carried out by testing the residual enzyme activity after the enzyme was incubated in 200 mM Na_2_HPO_4_-citrate buffer (pH 7.0) at 4 °C for 1 h in the presence of various metal compounds at a concentration of 2 mM and surfactants at a concentration of 5 mM. The mixture without any metal ion was used as the control with the corresponding enzyme activity designated as 100%. The influences of NaCl on Alg17B activity on the enzyme activity were performed by incubating the enzyme at the concentrations of 0, 0.25%, 0.50%, 0.75% and 1% (w/v) at 4 °C for 1 h. Further residual activities were then tested. Relative enzyme activity (100%) is defined as enzyme activity without addition of any ions.

### Alg17B reaction kinetic parameters

Concentrations of the SA substrate (0.3%, 0.4%, 0.5%, 0.6%, 0.7%, 0.8%, 0.9%, and 1%) were prepared with 50 mM Tris–HCl (pH 7.5) buffer, and the enzymatic activity at each concentration was determined. Using the Michaelis–Menten equation, the double reciprocal mapping method (Lineweaver and Burk 1934) was used to plot 1/*V* vs. 1/[*S*], and the kinetic parameters, *K*_m_ and *V*_max_, of alginate lyase were calculated.

### Substrate specificity

The substrate specificity of the enzyme was analyzed using SA, polyM and polyG respectively. 0.2% (w/v) of the above substrate was formulated with 50 mM Tris–HCl (pH 7.5) buffer and the enzyme activity was determined under optimal reaction conditions. PolyM and polyG were donated by the National Marine Medicine Engineering Technology Research Center of Ocean University of China.

### Alg17B hydrolysate analysis

Thin-layer chromatography (TLC) was performed to analyse the hydrolysed alginate lyase substrates, SA, polyM, and polyG. TLC was performed according to Dong et al. (2009) and TLC Silica gel 60 (Merck, Germany) was used as the thin chromatographic plate. Substrate solutions were prepared at a concentration of 1% (w/v). Pure enzyme solution was added to start the reaction under the optimum reaction conditions, and the reaction mixture sample was then applied to the plate. The developing agent was a mixture of 1-butanol alcohol, formic acid, and water at a ratio of 4:6:1 (v/v/v). Staining was performed using an ethanol solution containing 1% (v/v) sulfuric acid, and the colour was developed at 85 °C for 10 min.

### UHPLC-Q-Exactive analysis of enzyme degradation products

The purified Alg17B (2 mg/mL) was added into 2% (w/v) of SA, polyM, and polyG, respectively, and then incubated at 30 °C for 6 h. The resulting hydrolysates were concentrated and centrifuged to obtain supernatants for analysis. Subsequently, the acquired supernatants were filtered by 0.22 µm filter membrane, and analysed by a UltiMate 3000 UHPLC-Q-Exactive (Thermo, USA) mass spectrometer for identification of the enzyme degradation products. Liquid chromatography conditions were as follows: Column, ACQUITY UPLC HSS T3 C18, 2.1 mm × 100 mm, 1.8 μm (Waters, USA); mobile phase A, 0.1% formic acid; mobile phase B, acetonitrile; sample volume, 1 µL; flow rate, 0.4 mL/min; column temperature, 35 °C. Mass spectrometry conditions were listed as the following: Electrospray ion, HESI-II; spray voltage, 3.50 kv (+); shielding gas, N2, 30 L/min; auxiliary gas, N2, 10 L/min; temperature, 350 °C; temperature of ion transport tube, 320 °C; data acquisition, full scanning of primary ions in first-order spectrum and data-dependent scanning in second-order spectrum (Full MS/dd MS2); scanning mode, anion scanning; full scanning resolution, 70,000; maximum injection time, 100 ms; scanning range, 150–1700 m/z; secondary mass spectral resolution, 17,500; trigger threshold, 1.0e5; maximum injection time, 50 ms; normalized collision energy: 30, 60, 90.

## Results and discussions

### Phylogenetic tree and molecular biological identification of the BP-2 strain

**Fig. 1:**
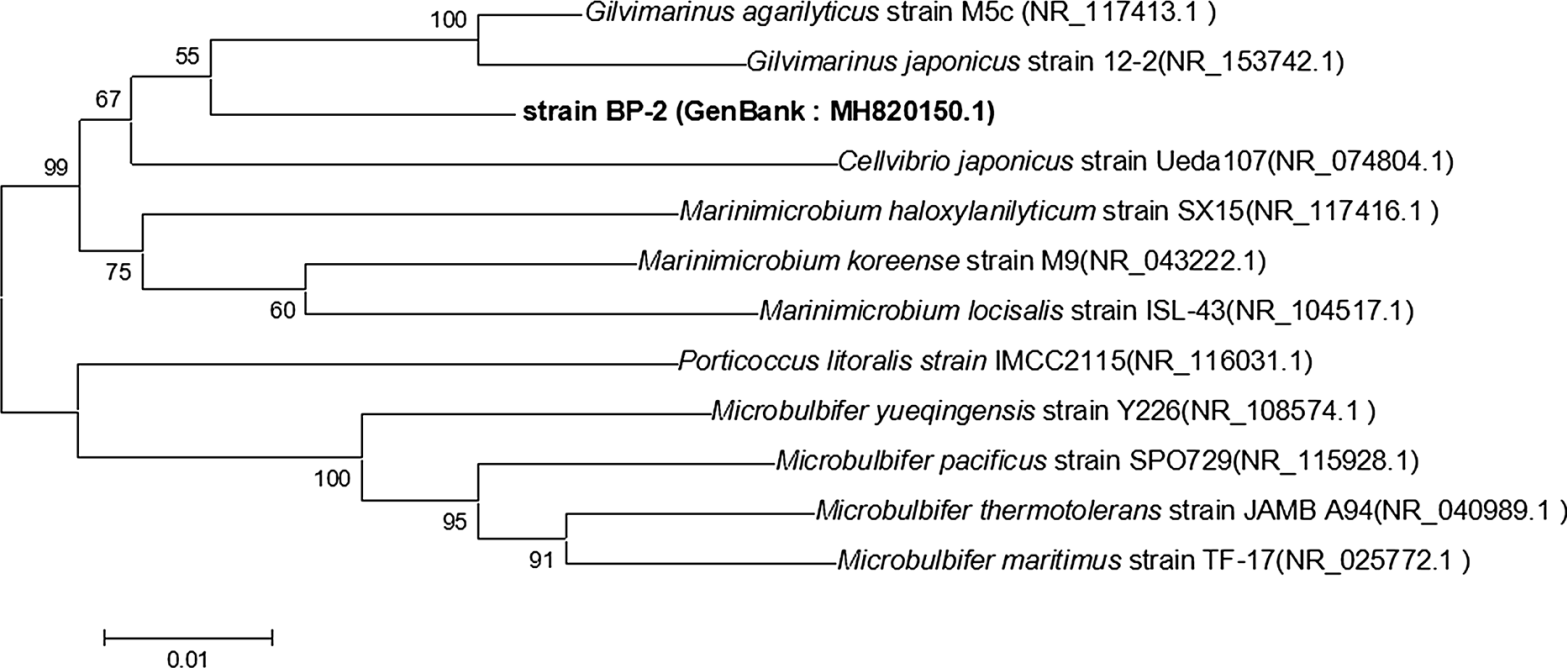
Phylogenetic tree of strain BP-2 based on 16S rDNA sequences. 16S rDNA sequences were aligned using ClustalX, and the phylogenetic tree was constructed using MEGA 5.1

Strain BP-2 producing the alginate lyase with the highest activity was screened from 24 isolates using alginate as the sole carbon source. The 16S rDNA sequence of the strain was cloned, sequenced, and submitted to GeneBank (accession number MH820150.1). A BLAST alignment of this 16S rDNA sequence was performed with the sequences in NCBI’s GenBank database (https://www.ncbi.nlm.nih.gov/), and the result showed that BP-2 was closely related to *Gilvimarinus agarilyticus*. *Gilvimarinus aglyphus* M5c (NR_117413.1) was its closest relative, with the highest similarity of 94%. The 11 strains most similar to BP-2 were selected for phylogenetic analysis. MEGA 5.1 (Xu et al. 2014) was used to construct a phylogenetic tree using the neighbour-joining (NJ) method (Fig. 1), and the results showed that the BP-2 strain was on an independent evolutionary branch. Combined with our previous analysis of morphological and physiochemical indexes (data not shown), the BP-2 strain may be a new species of marine bacteria.

### Alg17B isolation, purification and activity assay

The crude enzyme solution obtained from the concentration was applied to a DEAE Sepharose Fast Flow column for ion exchange chromatography. The NaCl gradient was employed for elution and the eluted fractions were tested for alginate lyase activity; the fractions with high enzyme activity were collected and concentrated for analysis. The purification factors and recoveries at each step in the enzyme purification process are shown in Table 1. The enzyme recovery rate after purification by freeze-drying, desalination by dialysis, and ion exchange chromatography was 24.87%; the purification factor reached 28.06-fold; and the specific activity of Alg17B after purification was 4036 U/mg. Compared with the activity of the overexpressed bifunctional alginate lyase reported by Huang et al. (2018) the Alg17B activity was near the activity of AlgM4 (4638 U/mg) and significantly higher than the activity of Aly1 (1261 U/mg) (Cheng et al. 2017). Thus, Alg17B may efficiently degrade alginate.

**Table 1:** Purification of alginate lyase Alg17B secreted by strain BP-2

Procedures	Total protein (mg)	Total activity (U)	Specific activity (U/mg)	Recovery (%)	Purification fold
Culture supernatant	2.94	424.81	145	100.00	1.00
Ammonium sulfate	0.13	324.55	2497	76.39	17.22
DEAE Sepharose FF	0.02	80.72	4036	24.87	27.83

**Fig. 2:**
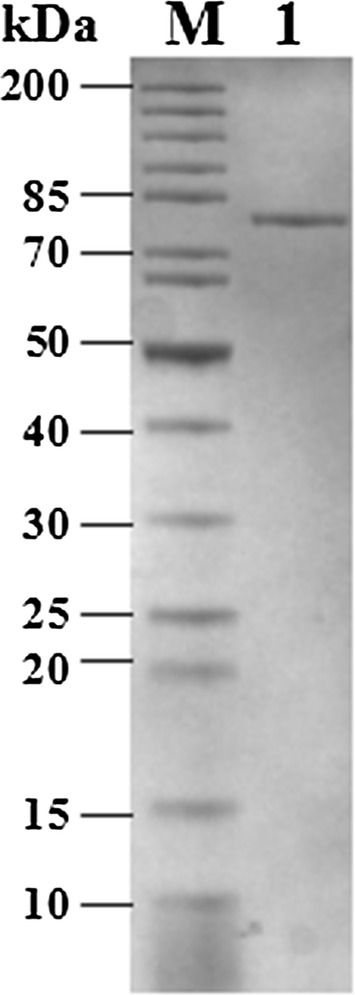
The SDS-PAGE analysis of purified Alg17B secreted by BP-2 strain. Lane M: molecular weight markers, Lane 1: purified Alg17B

SDS-PAGE of Alg17B after purification showed that the purified Alg17B had only one band with a molecular weight of approximately 77.0 kDa (Fig. 2). The molecular weight of Alg17B was smaller than those of alginate lyases reported to exhibit exolytic activity, such as OalB (83.0 kDa), OalC (81.0 kDa) (Ochiai et al. 2006), Alg17C (81.6 kDa) (Kim et al. 2012), and OalC17 (85.7 kDa) (Li et al. 2018).

### Alg17B sequence identification, analysis, and structural homology modelling

**Fig. 3:**
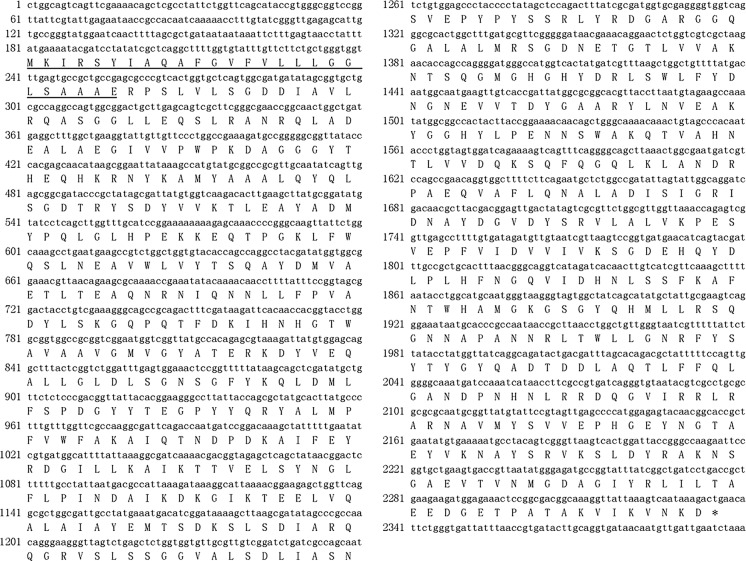
Nucleotide sequences of alginate lyase gene *alg17B* and its deduced amino acid sequences. The sequence of the signal peptide is underlined

Based on *N*-terminal amino acid sequencing of the purified Alg17B protein, the 12 amino acids arranged from the *N*-terminus were R P S L V L S G D D I A. After gene annotation analysis of genomic sequencing data of strain BP-2, the related coding genes for alginate lyase were identified. Based on the open reading frame (ORF) and signal peptide analysis of the coding genes (approximately 2 kb in size), the amino acid sequence of the protein was deduced from the gene sequence (Fig. 3). The alginate lyase gene was 2157 bp, encoding a protein of 718 amino acids. The encoding gene for the signal peptide was 78 bp, and the length of the encoded signal peptide was 26 amino acids.

**Fig. 4:**
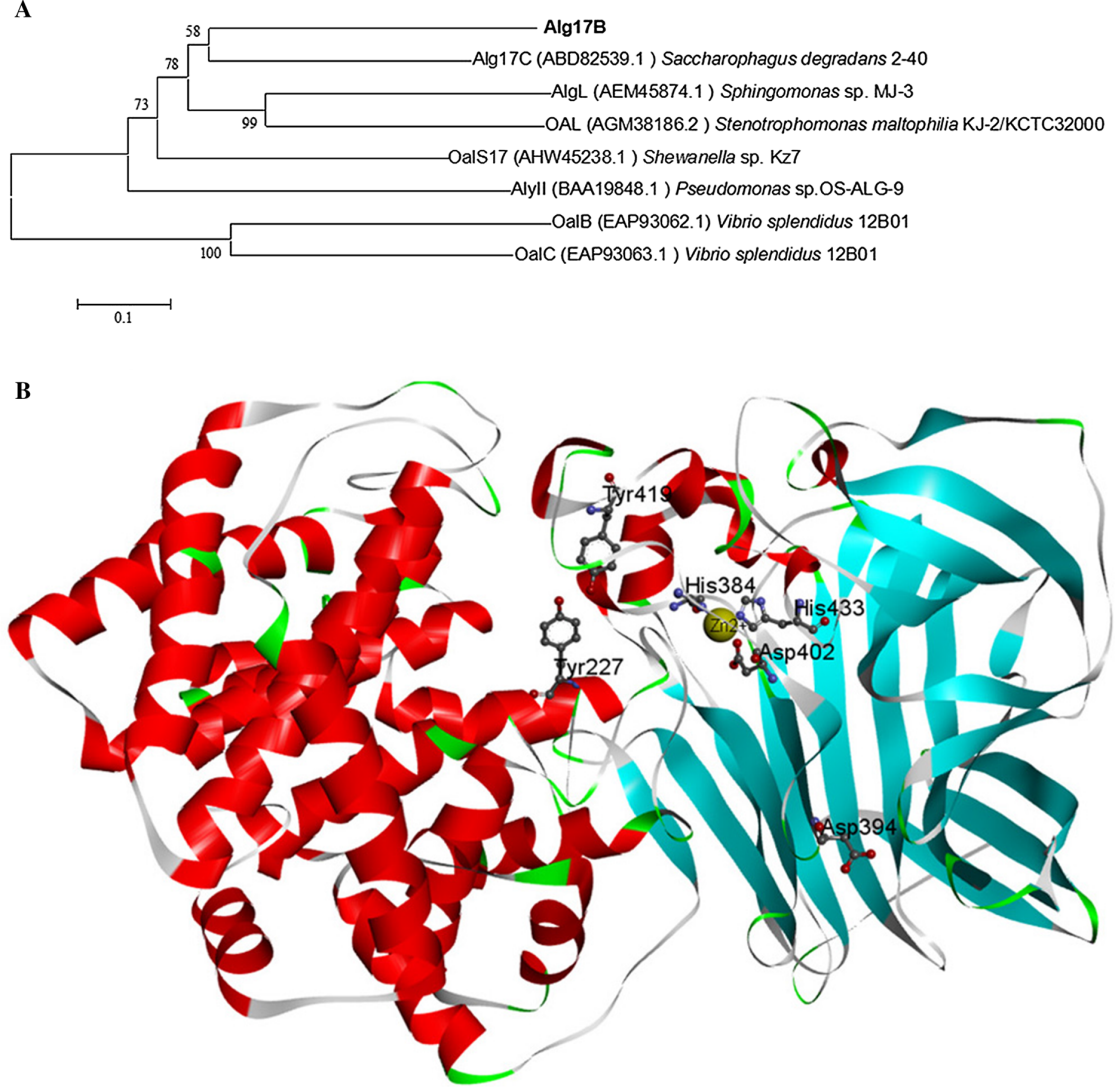
**a** Phylogenetic tree of Alg17B and other alginate lyases of PL17 family. Alginate lyase protein sequences were aligned using ClustalX, and the phylogenetic tree was constructed using MEGA 5.1 via the neighbor-joining method. **b** Model structure of Alg17B protein. The yellow ball represents a Zn^2+^ ion

In the CAZy (http://www.cazy.org/) database, alginate lyases are classified in the polysaccharide lyase (PL) protein family. The conserved domain of Alg17B, which was analysed by NCBI (https://www.ncbi.nlm.nih.gov/) Blast, showed that the Alg17B protein contains two domains: the *N*-terminal alginate lyase superfamily domain and the *C*-terminal heparinase II/III family protein domain. Some alginate lyases with exolytic activity, such as Atu3025, A1-IV, A1-IV, and MJ3-alginate lyase, also contain the heparinase II/III family protein domain, but whether this domain has catalytic activity remains unknown (Park et al. 2012). Alg17B was evolutionarily closest to Alg17C, the alginate lyase of *S*. *degradans* 2–40 (Fig. 4a), but their amino acid sequence similarity was only 52%. Alg17B could be a new alginate lyase in the PL17 family.

Protein Blast analysis showed that Alg17B has two possible catalytic sites, Tyr227 and Tyr419, as well as three substrate interaction sites, His384, Asp394, and His433, in a domain that is conserved in the PL17 protein family (Wang et al. 2015) (Fig. 4b). Among them, His384 and His433, plus Asp402 are assumed to be Zn^2+^-binding sites (Park et al. 2014) (Fig. 4b). Predicting tertiary structure is the focus of predicting protein structure and function. The homology modelling method was used to predict the tertiary structure of Alg17B. Using SWISS-MODEL software (Biasini et al. 2014), Alg17C (PDB code 4NEI) was automatically selected as the optimal template (Park et al. 2014). The sequence similarity between Alg17B and Alg17C was 52%, and a three-dimensional model was constructed based on this information. Discovery Studio 2016 software (Du et al. 2017) was used to generate the final tertiary structure (Fig. 4b). The results showed that the active site of Alg17B was in the α-helix region of the *N*-terminus, and the substrate binding site and Zn^2+^ ion-binding site were distributed in the β-sheet region of the *C*-terminus. The alginate lyase, *Tc*Alg1, is a macromolecular enzyme with exolytic activity, and both its active site and its substrate binding site lie in the α-helix of the *N*-terminus (Wang et al. 2018a), indicating the structural difference between Alg17B and *Tc*Alg1.

### Optimum temperature and thermal stability

**Fig. 5:**
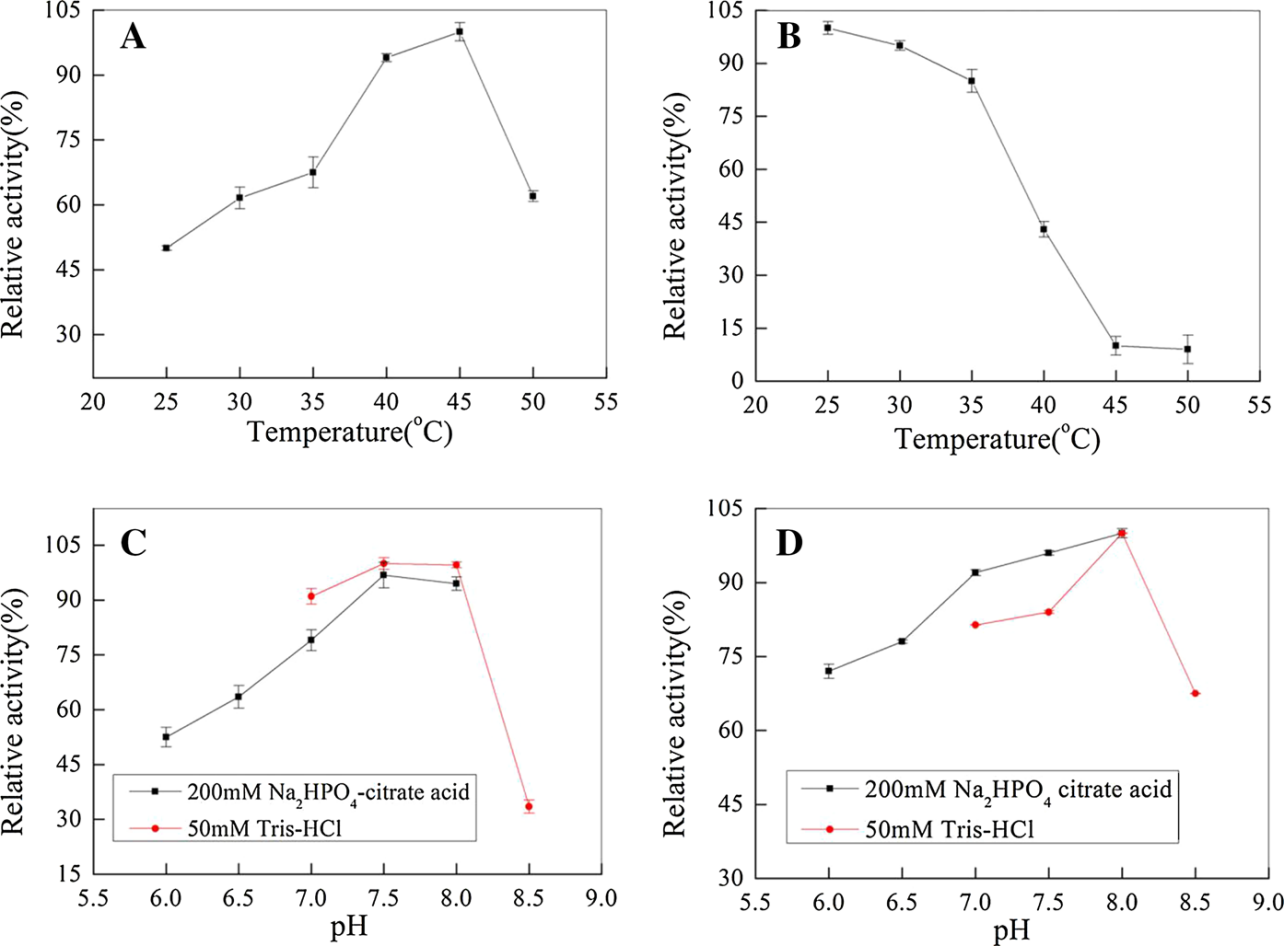
**a** Optimum temperature for purified Alg17B activity. The activity of Alg17B at 45 °C was completely retained. Data were shown as mean ± SD; n = 3. **b** Thermostability of purified Alg17B. The residual activity of Alg17B at 25 °C was completely retained. Data were shown as mean ± SD; n = 3. **c** Optimum pH for Alg17B activity,The activity of Alg17B in 50 mM Tris–HCl buffer (pH 8.0) was completely retained (Mean ± SD; n = 3). **d** pH stability of Alg17B. The residual activity of Alg17B in 50 mM Tris–HCl buffer or 200 mM Na_2_HPO_4_-citrate acid buffer (pH 8.0) was copmpletely retained (Mean ± SD; n = 3)

The activity of purified Alg17B was measured at different temperatures. Alg17B showed high activity at 40–45 °C, and 90% of the enzyme activity was retained at 40 °C, with the optimum temperature being 45 °C (Fig. 5a). The thermal stability of Alg17B was poor at 45 °C (Fig. 5b), and its residual activity was only 10%; however, its thermal stability was good at 25–35 °C, and 80% of its enzyme activity was retained in this temperature range. The stability of Alg17B rapidly decreased as the temperature increased.

### Optimum pH and pH stability

Alg17B activity was determined at different pH values. Its activity was highest when the pH was 7.5–8.0. This activity increased as the pH increased when the pH was below 7.5 and decreased as the pH increased when the pH was above 8.0 (Fig. 5c). The relative viability of the enzyme was reduced to 33% at pH 8.5. The pH stability test for Alg17B showed that the enzyme was stable at pH 7.5–8.0 (Fig. 5d), and more than 65% of the enzyme activity was retained at pH 6.0–8.5, exhibiting tolerance to different pH values.

### Effects of metal ions and surfactants on Alg17B activity

**Fig. 6:**
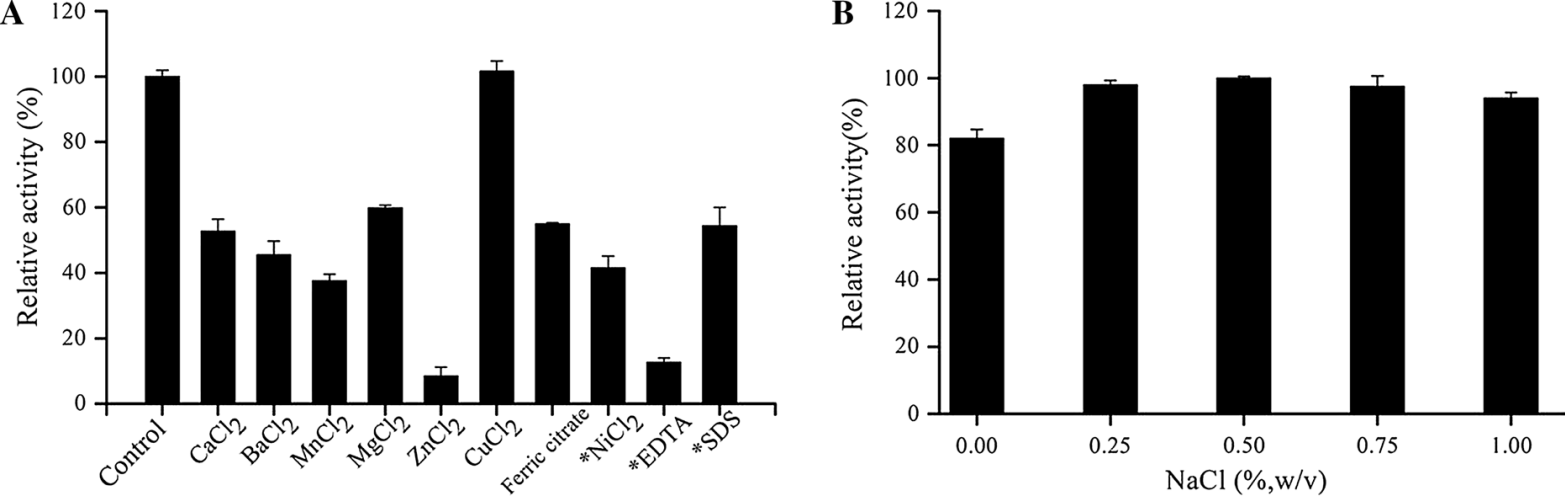
**a** Effect of chemicals on purified Alg17B activity. Data were shown as mean ± SD; n = 3. Asterisk: chemicals concentration is 5 mM; without Asterisk: 2 mM. **b** Effects of NaCl concentration on purified Alg17B activity. The highest activity of Alg17B with 0.5% (v/v) NaCl was set to be 100%. Data were shown as mean ± SD; n = 3

The effects of metal ions, ethylenediaminetetraacetic acid (EDTA) and sodium dodecyl sulphate (SDS) on Alg17B activity showed that Cu^2+^ did not affect Alg17B activity, while the other metal ions, Ca^2+^, Ba^2+^, Mn^2+^, Mg^2+^, Zn^2+^ and Ni^2+^, as well as the surfactants SDS and EDTA, significantly inhibited its activity (Fig. 6a). A low Na^+^ concentration significantly promoted Alg17B activity (Fig. 6b). In the concentration range of 0–0.5% (w/v) NaCl, Alg17B activity increased as the NaCl concentration increased; above 0.5% NaCl, the activity decreased as the NaCl concentration increased. When the concentration of NaCl reached 1%, 95% of the enzyme activity was retained.

One possible reason for the enhancement of enzyme activity by metal ions is that metal ions can act as cofactors at the enzyme’s active site to participate in the enzyme-catalysed reaction or decrease the ionic interactions between the substrates and enzymes by reducing the charge density on the substrate surface (Wong et al. 2000). Na^+^ can enhance the activity of the salt kinase AlyPM by enhancing the enzyme’s affinity to the substrate (Xiu-Lan et al. 2016) or enhance the activity of the salt kinase AlgM4 by altering the secondary structure. This structural change may be conducive to the enzyme and substrate binding and thus favourable to the enzymatic reaction (Huang et al. 2018).

AlyGC in the PL6 family are Ca^2+^-dependent alginate lyases, and Ca^2+^ can bind to the side chain of four acidic amino acids in these enzymes by linking atoms via coordinate bonds, thereby participating in binding the enzyme to the substrate and in the catalytic reactions (Xu et al. 2017). The substrate affinity of the alginate lyase AlgAT0, which is also a member of the PL6 family, can be improved via tightly binding Asp238 to Ca^2+^, thus favouring the enzymatic reaction (Wang et al. 2018b). Ca^2+^ at a concentration of 2 mM is required for the exolytic activity of the alginate lyase AlyA5 (Thomas et al. 2013); however, analysing the effect of Ca^2+^ on Alg17B activity showed that 2 mM Ca^2+^ significantly inhibited Alg17B activity by approximately 50%. Ca^2+^ may act as a noncompetitive inhibitor by noncompetitively binding to chemical groups other than the Alg17B active site to form a stable intermediate complex of the enzyme, the substrate and the noncompetitive inhibitor. This intermediate complex cannot be decomposed and inhibits the enzyme’s activity.

In the crystal structure of Alg17C, the His415, Asp433, and His464 side chains form a Zn^2+^-binding site (Park et al. 2014). After replacing the mutant H415A in His415 with Ala, the *k*_cat_/*K*_m_ value was nearly one thousand times lower than that of the wild-type protease (Park et al. 2014) and the catalytic activity of H415A was severely inhibited, indicating that His415 is closely related to the alginate protein catalytic activity of Alg17C. For the alginate lyase Alg17B, 2 mM Zn^2+^ severely inhibited the enzyme activity, which was only 8.47% of the original enzyme activity. In the Alg17B protein structure, His384, His433, and Asp402 are the Zn^2+^-binding sites, and His384 and His433 are also substrate binding sites. Zn^2+^ may serve as a competitive inhibitor by competing with the substrate for the same binding site in the Alg17B structure, thereby blocking the substrate from binding to the enzyme, greatly reducing the enzyme’s affinity for the substrate, and strongly inhibiting the enzyme activity.

### Enzyme reaction kinetics

SA solution was used as the substrate to study Alg17B’s catalytic reaction kinetics. The double reciprocal method was used to calculate the *K*_m_ and *V*_max_ of the enzyme. The calculation results showed that the *V*_max_ of alginate degradation by the BP-2 strain alginate lyase was 0.92 mg/mL min, and the *K*_m_ was 0.39 mg/mL. Alg17B had a lower *K*_m_ than other exolytic lyases, such as OacA (3.25 mg/mL), OalB (0.76 mg/mL), OalC (0.53 mg/mL), and Alg17C (35.2 mg/mL) (Kim et al. 2012; Ochiai et al. 2006), indicating that Alg17B has a high affinity for these substrates.

### TLC and UHPLC-Q-Exactive analysis of enzyme degradation products

**Fig. 7:**
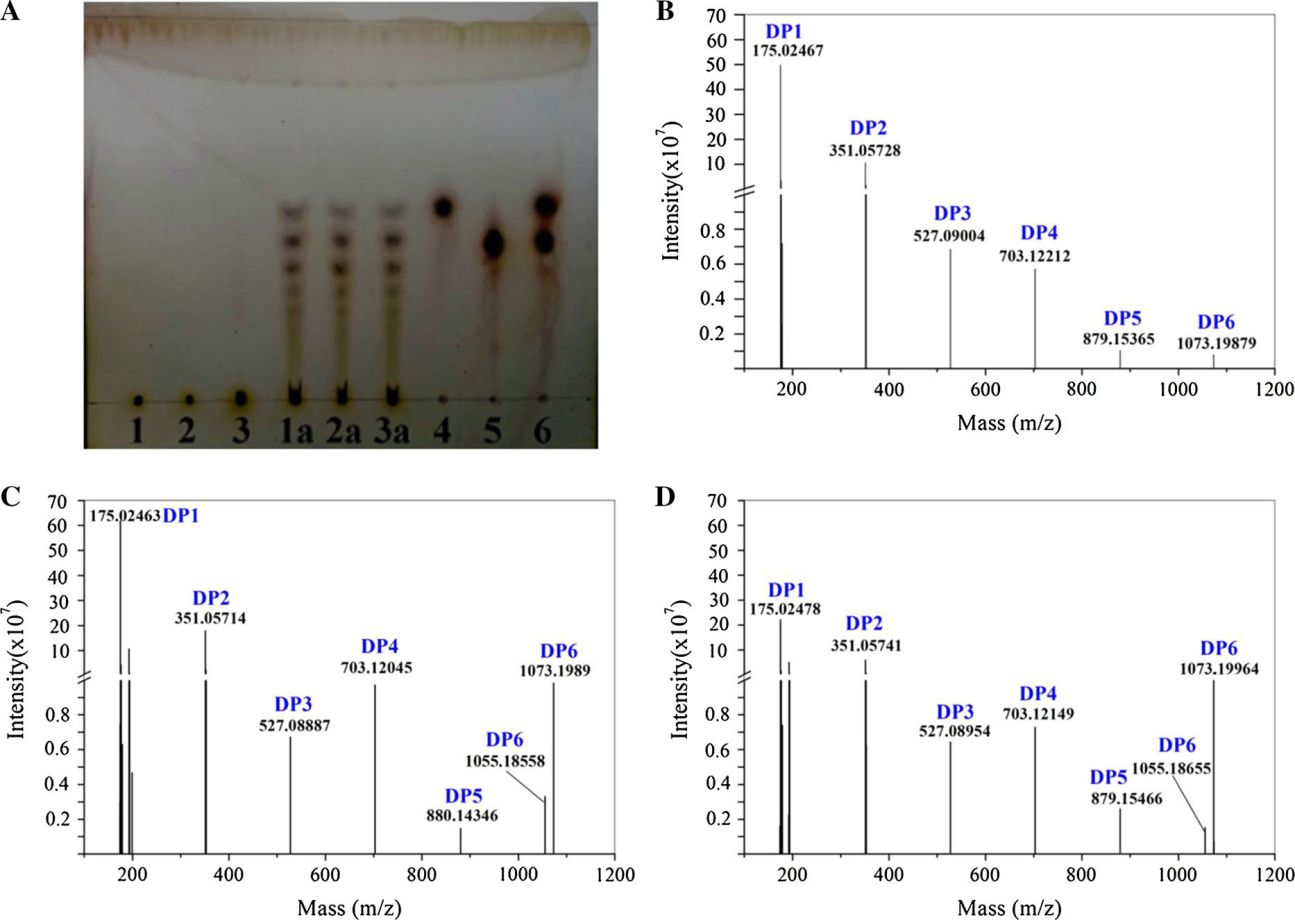
**a** TLC and **b**–**d** UHPLC-Q-Exactive analysis of the oligomers released from SA, polyM, and polyG by purified Alg17B from BP-2. **a** Lane 1: SA; Lane: PolyM; Lane 3: PolyG; Lane 1a: Reaction products generated from SA; Lane 2a: Reaction products generated from polyM; Lane 3a: Reaction products generated from polyG; Lane 4: Glucose; Lane 5: Maltose; Lane 6: The mixture of glucose and maltose. The main final products obtained from SA (**b**), polyM (**c**) and polyG (**d**) are monosaccharides (DP1)

The alginate lyase Alg17B produced by BP-2 is active against SA, polyM, and polyG substrates (Table 2). The polyM activity was relatively high, showing Alg17B’s preference for enzymatic hydrolysis of polyM. TLC analysis of the enzymatic hydrolysates showed that Alg17B directly hydrolysed the SA, polyM, and polyG substrates to produce monosaccharides, disaccharides, and other oligosaccharides (Fig. 7a). To further analyze the enzymatic hydrolysis products, high resolution mass spectrometry was applied to determine the precise molecular weight of the products generated by degradation of SA, polyM, and polyG, respectively. Alg17B was capable of breaking down the above three substrates to produce oligosaccharides with DP of 2–6 and the main products were monosaccharides, followed by disaccharides, whereas the content of the fractions with DP of 3–6 was relatively low (Fig. 7b–d). The monosaccharides derived from alginate could be used as fermental sugars for microbial production of bioenergy, and alginate disaccharides are considered powerful antioxidants with high medical value (Li et al. 2017). The degradation pattern of alginate was well documented in *Sphingomonas* sp. A1 (Takase et al. 2010), in which the alginates were depolymerized into oligosaccharides by endolytic alginate lyases, A1-I, A1-II, and A1-III. The oligosaccharides were then saccharified into monomers by exolytic alginate lyases A1-IV. Considering the dominance of monosaccharides and disaccharides in the hydrolysates, it can be inferred that Alg17B possesses both exolytic and endolytic activities toward the tested substrates with high efficiency. It is suggested that the saccharification of alginate for biofuel production requires synergistic effects of alginate lyases with endo- and exo- action modes. The dual action modes of Alg17B are advocated for saccharifying alginate to yield abundant monosaccharides for microbial fermentation of biofuels. Collectively, Alg17B revealed novelty and great potential in producing AOS with low DP, providing raw materials for industrial production of medicines, biofuels, and other biochemicals.

**Table 2:** Substrate specificity of purified Alg17B toward SA, polyM and polyG

Substrates	Relative activity (%)
SA	100.00 ± 0.01
PolyG	50.29 ± 0.33
PolyM	125.96 ± 0.55

The activity for degradation of SA was taken as 100%. Data were shown as mean ± SD; n = 3

## Conclusions

In this study, the alginate lyase, Alg17B, produced by the strain BP-2, was purified and characterized. Alg17B, which has a molecular weight of approximately 77 kDa, belongs to the PL17 family, exhibiting both endolytic and exolytic activities. The specific activity of the purified enzyme reached 4036 U/mg, with a smaller *K*_m_ value than other exolytic alginate lyases. Alg17B degrades SA, polyM, and polyG and can directly hydrolyse these substrates into monosaccharides that can be directly utilized by ethanol-fermenting microorganisms. This enzyme could serve as a new and efficient tool for saccharifying brown algae for ethanol production. Apart from monosaccharides, Alg17B produces oligosaccharides with DP of 2–6 from alginate, which can be applied in studying the preparation and biological activity of AOS.
